# Real-World Evidence for the Safety and Effectiveness of Naldemedine in the Management of Opioid-Induced Constipation in Patients With Cancer Pain: Post-hoc Subgroup Analysis of Post-marketing Surveillance in Japan

**DOI:** 10.7759/cureus.46090

**Published:** 2023-09-27

**Authors:** Noriyuki Naya, Hiroaki Oka, Sayo Hashimoto, Yasuhide Morioka, Yoshiyuki Kizawa

**Affiliations:** 1 Medical Affairs Department, Shionogi & Co. Ltd., Osaka, JPN; 2 Pharmacovigilance Department, Shionogi & Co. Ltd., Osaka, JPN; 3 Department of Palliative and Supportive Care, Faculty of Medicine, University of Tsukuba, Tsukuba, JPN

**Keywords:** post-marketing surveillance, opioid-induced constipation, opioid, naldemedine, cancer

## Abstract

Background: Opioid-induced constipation is common and greatly affects the quality of life but is often under-recognised and undertreated. This study aimed to investigate the safety and effectiveness of naldemedine for opioid-induced constipation with cancer pain according to specific subgroups of clinical interest.

Methods: In this exploratory post-hoc subgroup analysis of post-marketing surveillance from Japan (UMIN: 000042851), data were investigated by the subgroups: age (≥75, <75 years), Eastern Cooperative Oncology Group performance status (PS 0-2, 3-4), constipation severity (mild, moderate, severe), brain metastasis (yes, no), anticancer drug treatment (yes, no), opioid at naldemedine initiation (fentanyl only, only strong opioids other than fentanyl, weak opioids only, other), and prior or concomitant use of laxative (only osmotic/saline laxatives, only stimulant laxatives, other, none). Enrolled patients (n = 1184) received naldemedine (0.2 mg once daily) orally for up to 12 weeks. Regarding safety endpoints, the incidence of adverse drug reactions, including diarrhoea, was determined within each subgroup. Regarding effectiveness endpoints, improvement rates in the frequency and condition of bowel movements were investigated by subgroups.

Results: The incidence of adverse drug reactions, including diarrhoea, among subgroups ranged from 7.74% to 16.08% (diarrhoea: 5.95% to 13.19%), compared to 11.30% (diarrhoea: 9.09%) in the total population. Through week two to week 12, improvement rates in the frequency and condition of bowel movement among subgroups ranged from 63.6% to 89.7% and 67.6% to 94.9%, compared to 75.0% to 83.2% and 80.0% to 88.0% in the total population, respectively.

Conclusions: Naldemedine was well tolerated and effective in patients with opioid-induced constipation and cancer pain regardless of the subgroups investigated.

## Introduction

Opioid-induced bowel dysfunction (OIBD) is common among patients with moderate-to-severe chronic or cancer pain treated with opioids [1]. Opioid-induced constipation (OIC) is the most common form of OIBD, with a reported incidence of 51-87% in international studies and 56% in a Japanese observational cohort study [2,3]. Despite being common and having a major impact on quality of life [4], OIC is often under-recognised and undertreated by healthcare professionals and therefore represents an unmet treatment need among opioid-treated patients [4].

Pharmacological management of OIC as recommended by treatment guidelines follows a stepwise approach [2,5-7]. The American Gastroenterological Association guidelines on the medical management of OIC recommend traditional laxatives as first-line agents [7]. Similarly, Japanese Society for Palliative Medicine guidelines recommend the use of traditional agents prophylactically in patients at risk of OIC [6]. Peripherally acting µ-opioid receptor antagonists (PAMORA), which specifically block µ-opioid receptors in the gastrointestinal tract but do not cross the blood-brain barrier (BBB) and hence spare the analgesic effect of opioids, are recommended for patients with persistent constipation [7-10].

Naldemedine, a potent PAMORA [11], has an enhanced ability to resist transfer across the BBB due to the increased molecular weight and polarity of the molecule [12], which reduces potential suppressive effects on opioid analgesia [13,14]. Naldemedine has been approved in Japan, the US, the European Union, the UK, and Taiwan [15-17]. The efficacy and safety of naldemedine were confirmed in seven clinical trials, COMPOSE-1-7, for OIC in adults with cancer pain or chronic non-cancer pain [18-21]. COMPOSE-4, a randomised, double-blind, placebo-controlled trial in 193 patients with cancer pain, showed a significantly higher proportion of response (≥3 complete spontaneous bowel movements in a week) in naldemedine recipients than placebo recipients [19]. COMPOSE-5, an open-label, 12-week observational extension study of COMPOSE-4 found that approximately 80% of patients had at least one adverse event (AE), of which diarrhoea was the most common [19].

In Japan, a recent prospective post-marketing surveillance (PMS) examined the safety and effectiveness of naldemedine in routine clinical practice among patients with OIC and cancer pain [22]. As noted previously, AEs overall were common (64.23% of patients) but treatment-related AEs (adverse drug reactions (ADRs)) were infrequent (11.30% of patients), generally non-serious and tended to resolve within two weeks. Diarrhoea, again the most common ADR, was not influenced by baseline characteristics in terms of development or aggravation [22].

Against this background, this post-hoc analysis of the primary PMS in Japan sought to further investigate the safety and effectiveness of naldemedine in routine clinical practice in patients with OIC and cancer pain among subgroups of clinical interest. The subgroups to be investigated in this study were selected based on the special clinical interests of healthcare professionals, also considering the rationale that patients in routine clinical practice have a greater number of complications or concomitant medications than those in clinical trials [22]. In particular, it has been previously noted that there is a lack of information on the influence of concomitant opioids and laxative use on naldemedine [23], and hence, clarification of this influence is a key aim of this PMS post-hoc analysis. Given that naldemedine is recommended as part of a stepwise strategy in treatment guidelines, it is also of interest whether the initial severity of constipation affects the effectiveness or tolerability of naldemedine. We also chose to clarify the effect of brain metastases because BBB disruption is one of the conditions that might affect the efficacy and safety of naldemedine [19,24,25]. The subgroup analysis also sought to clarify the (i) incidence of ADRs, including diarrhoea, important identified risks in the risk management plan for naldemedine, and a specific ADR of interest in relation to background factors [22]; (ii) improvement rates in the frequency and condition of bowel movement.

## Materials and methods

Study design

An exploratory post-hoc subgroup analysis was conducted using a dataset of a prospective PMS conducted at 269 hospitals and clinics in Japan between January 2018 and June 2020 (UMIN registry no.: 000042851) [22].

Detailed methods of the original PMS have been published previously [22]. In brief, patients (n = 1184) with OIC and cancer pain who had never been treated with naldemedine were enrolled and administered naldemedine 0.2 mg once daily for up to 12 weeks. Surveillance data were recorded at two, four, eight, and 12 weeks after initiation or at discontinuation/completion of naldemedine treatment. The surveillance data on individual patients relevant to this exploratory subgroup analysis included the following: patient background factors; opioid-related and laxative-related variables such as administration route, dose, and treatment period for opioids or laxatives received from two weeks before naldemedine treatment to the end of naldemedine treatment; and non-opioid concomitant drug-related variables, including route of administration, dose, and treatment period. ADRs were defined as adverse events for which causality could not be excluded that developed after the initiation of naldemedine treatment. Regarding the effectiveness of naldemedine, qualitative evaluation based on patient interviews assessed (i) improvement in frequency of bowel movement (improved, unchanged, or worsened) and (ii) improvement in the condition of bowel movement (improved, slightly improved, unchanged, slightly worsened, or worsened), including stool hardness, straining, and sensation of incomplete evacuation at each evaluation.

This post-hoc analysis investigated the following subgroups in relation to the patient background, safety, and effectiveness: age (≥75, <75 years); Eastern Cooperative Oncology Group performance status (PS 0-2, PS 3-4); constipation severity (mild, moderate, severe); brain metastasis (yes, no); anticancer drug treatments, including antibody therapy and chemotherapy (yes, no); opioid use at the start of naldemedine treatment (fentanyl only, only strong opioids other than fentanyl, weak opioids only, others); and prior or concomitant use of laxative (only osmotic/saline laxatives, only stimulant laxatives, others, none). The age cut-off was selected as 75 years or older and is deemed latter-stage elderly in Japan [26]. Performance status (PS) groups were chosen as patients with advanced cancer who have impaired performance have been shown to have a higher prevalence of constipation and PS 3-4 was not included in previous clinical trials of naldemedine [27]. The severity of constipation was chosen as a subgroup to assess differences across this spectrum, especially since many cancer patients who receive opioids report moderate to severe constipation [4]. The presence or absence of brain metastasis was of interest given that their presence is a potential cause of BBB disruption, which is relevant to the mechanism of action of naldemedine [11]. Anticancer drug treatments were chosen as a simple categorical variable due to the possibility of chemotherapy-induced constipation or diarrhoea, which may act as a confounder of treatment effectiveness or tolerability [28]. The opioid class was selected based on literature showing differences in adverse event profiles, including in relation to constipation, among different opioid types [3,4]. Finally, prior or concomitant use of laxatives was of obvious interest given the potential influence of these agents on the overall efficacy of naldemedine and to explore differences between the main classes of laxative agents [29].

The original PMS was conducted in accordance with the Declaration of Helsinki and in compliance with Good Post-marketing Study Practice according to the ordinance by the Japanese Ministry of Health, Labour, and Welfare. According to the Good Post-marketing Study Practice Ordinance by the Ministry of Health, Labour, and Welfare, institutional review board approval and informed consent are not required in Japan [22].

Statistical analysis

The incidence of patient background factors and ADRs (including diarrhoea as a specific ADR of interest) were calculated as the ratio of cases versus the total number of patients for each subgroup. We defined “improved” as an improvement in the frequency of bowel movements and both “improved” and “slightly improved” as an improvement in the condition of bowel movements. The incidence of adverse drug reactions was rounded and displayed to the second decimal place. Ratios other than the above were rounded and displayed to the first decimal place.

For safety and effectiveness variables, the 95% confidence interval (95% CI) of the ratio and the chi-square test of independence were performed for each subgroup and, in the case of effectiveness, the observation period.

## Results

Patient background factors

Patient demographics, baseline characteristics, and treatment factors by subgroups for the safety analysis set are shown in Table 1, with corresponding data for the effectiveness analysis set shown in Supplementary Table A1. In general, there were no remarkable differences among the subgroups investigated. Several differences among corresponding subgroups were noted. The proportion of patients with PS 3-4 was 29.4% in patients ≥75 years and 37.2% in patients <75 years; 47.6% and 31.2% in patients with and without brain metastasis, respectively. Also, the proportion of patients with PS 3-4 ranged from 22.0% to 41.3% among subgroups related to opioid analgesics used when naldemedine was started. In addition to the differences noted above, patients with greater impairment (PS 3-4) were also more likely to be hospitalised (82.2% vs. 61.5% for patients with PS 0-2), have severe constipation (30.4% vs. 20.6% for patients with PS 0-2), and have non-cancer complications (70.1% vs. 61.4% for patients with PS 0-2). Finally, the proportion of patients hospitalised ranged from 60.1% to 77.3% among subgroups related to opioid analgesics used when naldemedine was started.

**Table 1:** Baseline characteristics of patients according to safety analysis set GI, gastrointestinal; OIC, opioid-induced constipation; SD, standard deviation.

Baseline characteristics	Age, years	Weight, kg	Sex, male/female	Hospitalised	OIC present	Severity of constipation			Performance status		Liver failure	Renal failure	Non-cancer complications	History of GI disease
						Mild	Moderate	Severe	0-2	3-4				
Subgroup	Mean (SD)	Mean (SD)	n/n (%/%)	n (%)	n (%)	n (%)	n (%)	n (%)	n (%)	n (%)	n (%)	n (%)	n (%)	n (%)
Total population	69.0 (12.8)	53.7 (11.3)	672/505 (57.1/42.9)	802 (68.1)	1040 (88.4)	233 (19.8)	527 (44.8)	280 (23.8)	795 (67.5)	381 (32.4)	114 (9.7)	74 (6.3)	755 (64.1)	220 (18.7)
Age (years)														
<75	61.7 (10.5)	55.3 (11.4)	406/322 (55.8/44.2)	487 (66.9)	640 (87.9)	152 (20.9)	309 (42.4)	179 (24.6)	514 (70.6)	214 (29.4)	72 (9.9)	40 (5.5)	450 (61.8)	122 (16.8)
≥75	80.9 (4.6)	51.0 (10.4)	266/183 (59.2/40.8)	315 (70.2)	400 (89.1)	81 (18.0)	218 (48.6)	101 (22.5)	281 (62.6)	167 (37.2)	42 (9.4)	34 (7.6)	305 (67.9)	98 (21.8)
Performance status														
0-2	68.0 (12.9)	54.0 (11.2)	459/336 (57.7/42.3)	489 (61.5)	690 (86.8)	186 (23.4)	340 (42.8)	164 (20.6)	795 (100)	0 (0)	73 (9.2)	48 (6.0)	488 (61.4)	152 (19.1)
3-4	71.2 (12.3)	53.0 (11.5)	212/169 (55.6/44.4)	313 (82.2)	349 (91.6)	47 (12.3)	186 (48.8)	116 (30.4)	0 (0)	381 (100)	41 (10.8)	26 (6.8)	267 (70.1)	68 (17.8)
Severity of constipation before naldemedine dosing														
Mild	68.2 (13.0)	54.4 (11.8)	140/93 (60.1/39.9)	160 (68.7)	233 (100.0)	233 (100.0)	0 (0.0)	0 (0.0)	186 (79.8)	47 (20.2)	26 (11.2)	11 (4.7)	149 (63.9)	41 (17.6)
Moderate	69.6 (12.7)	53.6 (10.8)	292/235 (55.4/44.6)	374 (71.0)	527 (100.0)	0 (0.0)	527 (100.0)	0 (0.0)	340 (64.5)	186 (35.3)	53 (10.1)	36 (6.8)	353 (67.0)	110 (20.9)
Severe	68.4 (13.3)	53.4 (11.6)	161/119 (57.5/42.5)	182 (65.0)	280 (100.0)	0 (0.0)	0 (0.0)	280 (100.0)	164 (58.6)	116 (41.4)	21 (7.5)	20 (7.1)	175 (62.5)	47 (16.8)
Brain metastasis														
No	69.4 (12.7)	53.7 (11.3)	622/471 (56.9/43.1)	737 (67.4)	967 (88.5)	222 (20.3)	485 (44.4)	260 (23.8)	751 (68.7)	341 (31.2)	110 (10.1)	71 (6.5)	698 (63.9)	207 (18.9)
Yes	63.9 (13.3)	53.5 (10.7)	50/34 (59.5/40.5)	65 (77.4)	73 (86.9)	11 (13.1)	42 (50.0)	20 (23.8)	44 (52.4)	40 (47.6)	4 (4.8)	3 (3.6)	57 (67.9)	13 (15.5)
Anticancer drug treatment														
No	70.4 (12.3)	52.7 (11.1)	408/339 (54.6/45.4)	504 (67.5)	659 (88.2)	134 (17.9)	344 (46.1)	181 (24.2)	476 (63.7)	270 (36.1)	72 (9.6)	46 (6.2)	465 (62.2)	137 (18.3)
Yes	66.6 (13.3)	55.3 (11.3)	264/166 (61.4/38.6)	298 (69.3)	381 (88.6)	99 (23.0)	183 (42.6)	99 (23.0)	319 (74.2)	111 (25.8)	42 (9.8)	28 (6.5)	290 (67.4)	83 (19.3)
Opioid analgesics used at naldemedine initiation														
Fentanyl only	68.3 (17.1)	50.2 (10.2)	44/31 (58.7/41.3)	58 (77.3)	69 (92.0)	18 (24.0)	29 (38.7)	22 (29.3)	44 (58.7)	31 (41.3)	8 (10.7)	3 (4.0)	46 (61.3)	15 (20.0)
Only strong opioids other than fentanyl	69.0 (12.3)	53.9 (11.3)	503/365 (57.9/42.1)	601 (69.2)	756 (87.1)	170 (19.6)	394 (45.4)	192 (22.1)	595 (68.5)	272 (31.3)	82 (9.4)	50 (5.8)	542 (62.4)	150 (17.3)
Weak opioids only	70.7 (13.0)	55.2 (9.5)	54/37 (59.3/40.7)	57 (62.6)	82 (90.1)	19 (20.9)	39 (42.9)	24 (26.4)	71 (78.0)	20 (22.0)	8 (8.8)	6 (6.6)	56 (61.5)	25 (27.5)
Others	68.4 (13.1)	52.9 (12.2)	71/72 (49.7/50.3)	86 (60.1)	133 (93.0)	26 (18.2)	65 (45.5)	42 (29.4)	85 (59.4)	58 (40.6)	16 (11.2)	15 (10.5)	111 (77.6)	30 (21.0)
Previously used laxatives														
Osmotic/saline laxatives only	69.3 (12.7)	53.6 (11.4)	234/195 (54.5/45.5)	276 (64.3)	402 (93.7)	91 (21.2)	233 (54.3)	78 (18.2)	297 (69.2)	132 (30.8)	30 (7.0)	23 (5.4)	272 (63.4)	70 (16.3)
Stimulant laxatives only	69.9 (12.0)	52.6 (10.7)	63/58 (52.1/47.9)	86 (71.1)	113 (93.4)	23 (19.0)	69 (57.0)	21 (17.4)	80 (66.1)	41 (33.9)	10 (8.3)	6 (5.0)	84 (69.4)	25 (20.7)
Others	68.7 (12.6)	53.5 (11.2)	167/137 (54.9/45.1)	227 (74.7)	295 (97.0)	54 (17.8)	168 (55.3)	73 (24.0)	181 (59.5)	123 (40.5)	40 (13.2)	24 (7.9)	226 (74.3)	79 (26.0)
Nothing	68.7 (13.4)	54.5 (11.3)	208/115 (64.4/35.6)	213 (65.9)	230 (71.2)	65 (20.1)	57 (17.6)	108 (33.4)	237 (73.4)	85 (26.3)	34 (10.5)	21 (6.5)	173 (53.6)	46 (14.2)
Concomitant laxatives														
Osmotic/saline laxatives only	69.5 (12.7)	53.9 (11.6)	235/195 (54.7/45.3)	282 (65.6)	389 (90.5)	97 (22.6)	213 (49.5)	79 (18.4)	300 (69.8)	130 (30.2)	27 (6.3)	16 (3.7)	256 (59.5)	60 (14.0)
Stimulant laxatives only	69.4 (13.0)	52.7 (10.5)	68/48 (58.6/41.4)	88 (75.9)	102 (87.9)	24 (20.7)	56 (48.3)	22 (19.0)	73 (62.9)	43 (37.1)	10 (8.6)	7 (6.0)	75 (64.7)	18 (15.5)
Others	68.6 (12.2)	53.5 (10.8)	232/172 (57.4/42.6)	297 (73.5)	379 (93.8)	64 (15.8)	211 (52.2)	104 (25.7)	262 (64.9)	141 (34.9)	52 (12.9)	34 (8.4)	303 (75.0)	100 (24.8)
Nothing	68.7 (13.9)	54.3 (11.8)	137/90 (60.4/39.6)	135 (59.5)	170 (74.9)	48 (21.1)	47 (20.7)	75 (33.0)	160 (70.5)	67 (29.5)	25 (11.0)	17 (7.5)	121 (53.3)	42 (18.5)

Safety

The incidence of ADRs, including diarrhoea as an ADR of special interest, for each subgroup is shown in Table 2. No notable differences in ADR incidence were observed within subgroups related to age, presence or absence of brain metastasis, opioid used at naldemedine initiation, and concomitant laxative use. The incidence of ADRs among the subgroups related to previously used laxatives was significantly different (P = 0.0191) with ADRs noted in 12.12%, 8.26%, 15.13%, and 7.74% of patients who received osmotic/saline laxatives only, stimulant laxatives/simulant laxatives only, others, and no previous laxative, respectively. The incidence of diarrhoea among the subgroups related to the severity of constipation was also significantly different (P = 0.0428) with ADRs noted in 8.58%, 11.39%, and 6.07% of patients who had mild, moderate, and severe constipation, respectively. The incidence of ADRs was significantly lower in patients with PS 3-4 than in patients with PS 0-2 (8.40% vs. 12.70%, respectively; P = 0.0291). The incidences of ADRs overall and diarrhoea were significantly greater in patients undergoing anticancer drug treatment compared with those who were not (ADRs overall, 15.58% vs. 8.84%, P = 0.0004; diarrhoea, 13.02% vs. 6.83%, P = 0.0004).

**Table 2:** Incidence of all adverse drug reactions and diarrhoea by subgroup CI, confidence interval

Category		Adverse drug reactions (ADR)		Diarrhoea (as ADR)	
		% (n/N)	95% CI	% (n/N)	95% CI
Total population		11.30 (133/1177)	9.547, 13.249	9.09 (107/1177)	7.510, 10.880
Age (years)	<75	12.64 (92/728)	10.310, 15.272	10.16 (74/728)	8.066, 12.593
	≥75	9.13 (41/449)	6.633, 12.184	7.35 (33/449)	5.113, 10.167
	P-value	0.0650		0.1027	
Performance status	0-2	12.70 (101/795)	10.468, 15.221	9.81 (78/795)	7.833, 12.094
	3-4	8.40 (32/381)	5.816, 11.650	7.61 (29/381)	5.157, 10.749
	P-value	0.0291		0.2196	
Severity of constipation before naldemedine dosing	Mild	13.30 (31/233)	9.222, 18.351	8.58 (20/233)	5.322, 12.946
	Moderate	13.28 (70/527)	10.503, 16.482	11.39 (60/527)	8.801, 14.411
	Severe	7.86 (22/280)	4.989, 11.654	6.07 (17/280)	3.576, 9.543
	P-value	0.0553		0.0428	
Brain metastasis	No	11.34 (124/1093)	9.525, 13.376	9.33 (102/1093)	7.673, 11.214
	Yes	10.71 (9/84)	5.018, 19.367	5.95 (5/84)	1.961, 13.347
	P-value	0.8603		0.2991	
Anticancer drug treatment	No	8.84 (66/747)	6.899, 11.104	6.83 (51/747)	5.125, 8.879
	Yes	15.58 (67/430)	12.284, 19.361	13.02 (56/430)	9.990, 16.576
	P-value	0.0004		0.0004	
Opioid analgesics used when naldemedine was started	Fentanyl only	8.00 (6/75)	2.993, 16.604	6.67 (5/75)	2.200, 14.876
	Only strong opioids other than fentanyl	10.37 (90/868)	8.420, 12.591	8.29 (72/868)	6.547, 10.332
	Weak opioids only	15.38 (14/91)	8.674, 24.464	13.19 (12/91)	7.004, 21.902
	Others	16.08 (23/143)	10.477, 23.150	12.59 (18/143)	7.634, 19.162
	P-value	0.0959		0.1604	
Previously used laxatives	Osmotic/saline laxatives only	12.12 (52/429)	9.187, 15.590	10.02 (43/429)	7.349, 13.263
	Stimulant laxatives only	8.26 (10/121)	4.034, 14.674	6.61 (8/121)	2.897, 12.612
	Others	15.13 (46/304)	11.296, 19.663	11.51 (35/304)	8.152, 15.647
	Nothing	7.74 (25/323)	5.071, 11.214	6.50 (21/323)	4.069, 9.767
	P-value	0.0191		0.1055	
Concomitant laxatives	Osmotic/saline laxatives only	11.40 (49/430)	8.550, 14.784	9.30 (40/430)	6.729, 12.452
	Stimulant laxatives only	7.76 (9/116)	3.609, 14.218	6.03 (7/116)	2.460, 12.038
	Others	14.11 (57/404)	10.864, 17.890	10.89 (44/404)	8.026, 14.344
	Nothing	7.93 (18/227)	4.767, 12.243	7.05 (16/227)	4.082, 11.194
	P-value	0.0655		0.2546	

Seriousness, time of onset, treatment, and outcome by type of ADR according to subgroups are summarised in Supplementary Table A2.

Effectiveness

Improvement rates in the frequency and condition of bowel movement by subgroups are shown in Table 3 and Table 4, respectively. No notable differences in improvement rates in either the frequency or condition of bowel movement were observed for subgroups related to age, PS, severity of constipation before naldemedine dosing, cancer treatment or opioid analgesics used when naldemedine was started. There was no clear association between the presence or absence of brain metastasis and improvement in frequency or condition of bowel movement despite a higher incidence of improvement in the condition of bowel movement only at four weeks in patients without brain metastasis (83.5% compared with 67.6% for patients with brain metastasis, P = 0.0181). Regarding previously used laxatives, significant differences among subgroups for improvement rates in frequency and condition of bowel movement were observed at two weeks (P = 0.0073 and P = 0.0066, respectively). There was also a significant difference only in the condition of bowel movement at 12 weeks (P = 0.0479). Regarding concomitant laxative use, there were significant differences in the improvement in the condition of bowel movement between concomitant laxative types at two weeks with improvement greatest in patients who received osmotic/saline laxatives only (P = 0.0176).

**Table 3:** Improvement rate in frequency of bowel movement by subgroups Note: Improvement rate was calculated as the proportion of patients with improvement in frequency of bowel movement divided by total patients (improved, unchanged, or worsened).

Category		2 weeks	4 weeks	8 weeks	12 weeks
		Improvement rate % (n/N)	Improvement rate % (n/N)	Improvement rate % (n/N)	Improvement rate % (n/N)
Total population		75.0 (642/856)	77.2 (461/597)	76.2 (323/424)	83.2 (228/274)
Age (years)	<75	74.4 (395/531)	78.3 (296/378)	77.8 (210/270)	82.5 (156/189)
	≥75	76.0 (247/325)	75.3 (165/219)	73.4 (113/154)	84.7 (72/85)
	P-value	0.5971	0.4052	0.3062	0.6572
Performance status	0-2	74.8 (436/583)	77.6 (330/425)	77.6 (260/335)	82.5 (188/228)
	3-4	75.4 (205/272)	76.0 (130/171)	70.5 (62/88)	86.7 (39/45)
	P-value	0.8548	0.6692	0.1611	0.4904
Severity of constipation before naldemedine dosing	Mild	68.8 (132/192)	71.6 (96/134)	69.6 (80/115)	78.5 (62/79)
	Moderate	77.3 (337/436)	78.4 (239/305)	81.1 (159/196)	86.4 (102/118)
	Severe	75.9 (173/228)	79.7 (126/158)	74.3 (84/113)	83.1 (64/77)
	P-value	0.0701	0.2050	0.0601	0.3419
Brain metastasis	No	74.9 (599/800)	77.6 (437/563)	75.8 (304/401)	83.1 (217/261)
	Yes	76.8 (43/56)	70.6 (24/34)	82.6 (19/23)	84.6 (11/13)
	P-value	0.7496	0.3425	0.4567	0.8897
Anticancer drug treatment	No	76.0 (402/529)	77.6 (266/343)	77.1 (165/214)	83.9 (115/137)
	Yes	73.4 (240/327)	76.8 (195/254)	75.2 (158/210)	82.5 (113/137)
	P-value	0.3937	0.8224	0.6522	0.7465
Opioid analgesics used when naldemedine was started	Fentanyl only	63.6 (35/55)	72.5 (29/40)	82.1 (23/28)	88.2 (15/17)
	Only strong opioids other than fentanyl	76.6 (480/627)	77.6 (340/438)	75.7 (237/313)	82.7 (158/191)
	Weak opioids only	69.5 (41/59)	73.7 (28/38)	71.4 (25/35)	77.8 (21/27)
	Others	74.8 (86/115)	79.0 (64/81)	79.2 (38/48)	87.2 (34/39)
	P-value	0.1354	0.8096	0.7394	0.7173
Previously used laxatives	Osmotic/saline laxatives only	79.8 (269/337)	80.9 (195/241)	78.9 (138/175)	83.2 (94/113)
	Stimulant laxatives only	72.0 (67/93)	69.4 (43/62)	71.4 (30/42)	81.3 (26/32)
	Others	75.9 (186/245)	79.4 (139/175)	80.5 (99/123)	89.7 (70/78)
	Nothing	66.3 (120/181)	70.6 (84/119)	66.7 (56/84)	74.5 (38/51)
	P-value	0.0073	0.0573	0.0835	0.1554
Concomitant laxatives	Osmotic/saline laxatives only	78.1 (257/329)	76.8 (182/237)	76.6 (131/171)	81.7 (89/109)
	Stimulant laxatives only	72.0 (59/82)	68.5 (37/54)	68.8 (22/32)	81.0 (17/21)
	Others	74.4 (244/328)	79.2 (187/236)	79.5 (140/176)	86.2 (100/116)
	Nothing	70.1 (82/117)	78.6 (55/70)	66.7 (30/45)	78.6 (22/28)
	P-value	0.2979	0.3967	0.2276	0.6954

**Table 4:** Improvement rate in the condition of bowel movement by subgroups Note: Improvement rate was calculated as the proportion of patients with improvement (either improved or slightly improved) in frequency of bowel condition divided by total patients (improved, slightly improved, unchanged, or worsened).

Category		2 weeks	4 weeks	8 weeks	12 weeks
		Improvement rate % (n/N)	Improvement rate % (n/N)	Improvement rate % (n/N)	Improvement rate % (n/N)
Total population		80.0 (685/856)	82.6 (493/597)	81.6 (346/424)	88.0 (241/274)
Age (years old)	<75	79.8 (424/531)	83.1 (314/378)	83.7 (226/270)	87.3 (165/189)
	≥75	80.3 (261/325)	81.7 (179/219)	77.9 (120/154)	89.4 (76/85)
	P-value	0.8707	0.6788	0.1395	0.6196
Performance status	0-2	80.6 (470/583)	83.3 (354/425)	83.3 (279/335)	88.2 (201/228)
	3-4	78.7 (214/272)	80.7 (138/171)	75.0 (66/88)	86.7 (39/45)
	P-value	0.5087	0.4507	0.0746	0.7791
Severity of constipation before naldemedine dosing	Mild	77.6 (149/192)	79.9 (107/134)	76.5 (88/115)	86.1 (68/79)
	Moderate	81.4 (355/436)	83.9 (256/305)	86.2 (169/196)	90.7 (107/118)
	Severe	79.4 (181/228)	82.3 (130/158)	78.8 (89/113)	85.7 (66/77)
	P-value	0.5235	0.5790	0.0681	0.4833
Brain metastasis	No	79.9 (639/800)	83.5 (470/563)	81.8 (328/401)	88.1 (230/261)
	Yes	82.1 (46/56)	67.6 (23/34)	78.3 (18/23)	84.6 (11/13)
	P-value	0.6816	0.0181	0.6705	0.7045
Anticancer drug treatment	No	81.3 (430/529)	83.4 (286/343)	83.2 (178/214)	89.1 (122/137)
	Yes	78.0 (255/327)	81.5 (207/254)	80.0 (168/210)	86.9 (119/137)
	P-value	0.2401	0.5481	0.3985	0.5776
Opioid analgesics used when naldemedine was started	Fentanyl only	72.7 (40/55)	75.0 (30/40)	78.6 (22/28)	94.1 (16/17)
	Only strong opioids other than fentanyl	81.3 (510/627)	83.6 (366/438)	81.5 (255/313)	86.9 (166/191)
	Weak opioids only	78.0 (46/59)	78.9 (30/38)	80.0 (28/35)	88.9 (24/27)
	Others	77.4 (89/115)	82.7 (67/81)	85.4 (41/48)	89.7 (35/39)
	P-value	0.3668	0.5240	0.8732	0.8143
Previously used laxatives	Osmotic/saline laxatives only	84.6 (285/337)	86.7 (209/241)	83.4 (146/175)	87.6 (99/113)
	Stimulant laxatives only	74.2 (69/93)	77.4 (48/62)	83.3 (35/42)	87.5 (28/32)
	Others	81.2 (199/245)	82.3 (144/175)	82.9 (102/123)	94.9 (74/78)
	Nothing	72.9 (132/181)	77.3 (92/119)	75.0 (63/84)	78.4 (40/51)
	P-value	0.0066	0.0967	0.3831	0.0479
Concomitant laxatives	Osmotic/saline laxatives only	84.5 (278/329)	84.4 (200/237)	83.0 (142/171)	86.2 (94/109)
	Stimulant laxatives only	75.6 (62/82)	77.8 (42/54)	81.3 (26/32)	85.7 (18/21)
	Others	79.6 (261/328)	81.4 (192/236)	81.8 (144/176)	91.4 (106/116)
	Nothing	71.8 (84/117)	84.3 (59/70)	75.6 (34/45)	82.1 (23/28)
	P-value	0.0176	0.6167	0.7197	0.4611

## Discussion

This post-hoc analysis of a PMS in Japan in patients with OIC and cancer pain found that both the safety and effectiveness of naldemedine overall were not remarkably different among the subgroups investigated. This concurs with the safety results of the primary PMS in which diarrhoea, the most common ADR for naldemedine, was not seemingly affected by patient characteristics except for a lower incidence in patients without complications or those who did not receive concomitant drugs other than opioids or laxatives.

In this post-hoc analysis, the incidence of diarrhoea was greater in patients receiving anti-cancer drugs at baseline, which seems intuitive, given the frequent association between these treatments and diarrhoea development [28]. Importantly, however, there was no negative influence of impaired performance on the safety of naldemedine.

Regarding effectiveness, the results of the primary PMS from which this post-hoc analysis is based also showed a relative lack of influence of baseline patient or treatment characteristics [22]. In the primary PMS, the proportion of patients with improvement in the condition of bowel movement was greater in patients who had previously used laxatives (90.1%) compared with those who had not (78.4%, P = 0.02) [22]. We speculate that this difference may stem from improvements in constipation related to factors other than OIC, which may also mean constipation remaining after treatment with laxatives is mostly OIC alone, and naldemedine might be more effective. Similar results were found in the present post-hoc analysis, with significant differences noted at various time points in the improvement rates in frequency and condition of bowel movement, which were greatest in patients who received concomitant osmotic/saline laxatives only. Further, patients already receiving these laxatives may have benefitted from them if the mechanism of constipation in these cases was unrelated to opioids. Regarding opioid type, fentanyl has been conventionally regarded as less constipating than other opioids due to avoidance of the oral route, reduction in first-pass metabolism, and other mechanisms despite any direct comparison studies [30]. The present analysis found no notable differences in naldemedine efficacy between fentanyl and other opioid groups studied, which potentially allows prescribing regardless of opioid type.

The present results are also supported by those of a pooled, subgroup analysis of two randomised, double-blind, placebo-controlled studies in a total of 307 Japanese patients with OIC and cancer pain [23]. In all subgroups examined, the incidence of diarrhoea was generally similar among patients within various subgroups, including those related to age, BMI, sex, opioid use, laxative use, and anticancer treatment. Further, it was previously reported that changes from baseline in Numerical Rating Scale (NRS) scores and Clinical Opioid Withdrawal Scale (COWS) scores were similar between patients who received naldemedine or placebo, regardless of potential BBB disruption [23]. In the present analysis, no effect of brain metastasis (as a possible cause of BBB disruption) was seen in relation to safety. Similarly, the effectiveness of naldemedine was noted in all subgroups investigated, although the proportion of responders was greater in patients who had received anticancer therapy.

Limitations and strengths

The main limitation of this study was the small number of patients within certain subgroups. Other limitations are consistent with those of the PMS upon which this subanalysis is based. These include the lack of placebo control, as well as potential biases from (i) the subjective methods to assess the condition and frequency of bowel movements, (ii) small sample sizes at certain time points due to treatment discontinuation related to cancer progression, and (iii) the involvement of a pharmaceutical company as a sponsor despite the analysis being based on a fixed analysis plan, as well as data input and results interpretation being handled by physicians at specific sites. Further, these results relate to patients in Japan, and generalizability to populations outside of Japan may be limited. Finally, this subanalysis was not powered to detect efficacy differences between treatment groups, so findings should be regarded as exploratory.

On the other hand, the results of this analysis are strengthened by the fact that the survey was conducted in a real-world setting and included patients who would be excluded from clinical studies. As such, practitioners can be more confident that these results relate to the types of patients they are likely to see in clinical practice.

## Conclusions

The results of post-hoc analysis of Japanese patients with OIC and cancer pain enrolled in a PMS for age, PS, constipation severity, brain metastasis, anticancer drug treatment, opioid at naldemedine initiation, and prior or concomitant use of laxative showed that naldemedine was well tolerated and effective. Naldemedine appeared to be a useful treatment option for patients with OIC and cancer pain, regardless of the investigated subgroups of clinical interest.

## Appendices

 Supplementary Table A1

**Table 5:** Baseline characteristics of patients according to the effectiveness analysis set GI, gastrointestinal; OIC, opioid-induced constipation; SD, standard deviation.

Baseline characteristic	Age, years	Weight, kg	Sex, male/female	Hospitalised	OIC present	Severity of constipation			Performance status		Liver failure	Renal failure	Non-cancer complications	History of GI disease
						Mild	Moderate	Severe	0-2	3-4				
Subgroup	Mean (SD)	Mean (SD)	n/n (%/%)	n (%)	n (%)	n (%)	n (%)	n (%)	n (%)	n (%)	n (%)	n (%)	n (%)	n (%)
Total population	68.9 (12.9)	53.5 (11.0)	543/410 (57.0/43.0)	656 (68.8)	953 (100.0)	211 (22.1)	485 (50.9)	257 (27.0)	645 (67.7)	307 (32.2)	91 (9.5)	56 (5.9)	622 (65.3)	182 (19.1)
Age (years)														
<75	61.6 (10.6)	54.9 (11.2)	332/259 (56.2/43.8)	406 (68.7)	591 (100.0)	141 (23.9)	284 (48.1)	166 (28.1)	414 (70.1)	177 (29.9)	56 (9.5)	32 (5.4)	371 (62.8)	99 (16.8)
≥75	80.9 (4.5)	51.1 (10.3)	211/151 (58.3/41.7)	250 (69.1)	362 (100.0)	70 (19.3)	201 (55.5)	91 (25.1)	231 (63.8)	130 (35.9)	35 (9.7)	24 (6.6)	251 (69.3)	83 (22.9)
Performance status														
0-2	67.9 (13.0)	53.9 (11.1)	375/270 (58.1/41.9)	406 (62.9)	645 (100.0)	173 (26.8)	322 (49.9)	150 (23.3)	645 (100.0)	0 (0)	58 (9.0)	37 (5.7)	401 (62.2)	129 (20.0)
3-4	70.9 (12.4)	52.6 (10.9)	167/140 (54.4/45.6)	250 (81.4)	307 (100.0)	38 (12.4)	162 (52.8)	107 (34.9)	0 (0)	307 (100.0)	33 (10.7)	19 (6.2)	221 (72.0)	53 (17.3)
Severity of constipation before naldemedine dosing														
Mild	68.0 (13.0)	54.3 (12.0)	125/86 (59.2/40.8)	143 (67.8)	211 (100.0)	211 (100.0)	0 (0.0)	0 (0.0)	173 (82.0)	38 (18.0)	25 (11.8)	7 (3.3)	134 (63.5)	36 (17.1)
Moderate	69.5 (12.8)	53.2 (10.2)	268/217 (55.3/44.7)	346 (71.3)	485 (100.0)	0 (0.0)	485 (100.0)	0 (0.0)	322 (66.4)	162 (33.4)	47 (9.7)	32 (6.6)	328 (67.6)	104 (21.4)
Severe	68.5 (12.8)	53.5 (11.6)	150/107 (58.4/41.6)	167 (65.0)	257 (100.0)	0 (0.0)	0 (0.0)	257 (100.0)	150 (58.4)	107 (41.6)	19 (7.4)	17 (6.6)	160 (62.3)	42 (16.3)
Brain metastasis														
No	69.3 (12.7)	53.6 (11.1)	506/383 (56.9/43.1)	605 (68.1)	889 (100.0)	201 (22.6)	449 (50.5)	239 (26.9)	611 (68.7)	277 (31.2)	88 (9.9)	53 (6.0)	578 (65.0)	173 (19.5)
Yes	63.0 (13.8)	52.2 (9.6)	37/27 (57.8/42.2)	51 (79.7)	64 (100.0)	10 (15.6)	36 (56.3)	18 (28.1)	34 (53.1)	30 (46.9)	3 (4.7)	3 (4.7)	44 (68.8)	9 (14.1)
Anticancer drug treatment														
No	70.4 (12.4)	52.3 (10.7)	329/268 (55.1/44.9)	406 (68.0)	597 (100.0)	122 (20.4)	315 (52.8)	160 (26.8)	381 (63.8)	215 (36.0)	59 (9.9)	34 (5.7)	378 (63.3)	112 (18.8)
Yes	66.5 (13.3)	55.4 (11.3)	214/142 (60.1/39.9)	250 (70.2)	356 (100.0)	89 (25.0)	170 (47.8)	97 (27.2)	264 (74.2)	92 (25.8)	32 (9.0)	22 (6.2)	244 (68.5)	70 (19.7)
Opioid analgesics used at naldemedine initiation														
Fentanyl only	67.3 (17.3)	49.6 (9.4)	36/28 (56.3/43.8)	48 (75.0)	64 (100.0)	17 (26.6)	27 (42.2)	20 (31.3)	39 (60.9)	25 (39.1)	7 (10.9)	3 (4.7)	41 (64.1)	10 (15.6)
Only strong opioids other than fentanyl	68.8 (12.4)	53.7 (11.0)	398/287 (58.1/41.9)	480 (70.1)	685 (100.0)	151 (22.0)	359 (52.4)	175 (25.5)	474 (69.2)	210 (30.7)	66 (9.6)	35 (5.1)	434 (63.4)	122 (17.8)
Weak opioids only	71.3 (13.1)	55.5 (9.7)	45/32 (58.4/41.6)	50 (64.9)	77 (100.0)	18 (23.4)	36 (46.8)	23 (29.9)	59 (76.6)	18 (23.4)	5 (6.5)	5 (6.5)	49 (63.6)	22 (28.6)
Others	68.9 (12.5)	52.8 (12.6)	64/63 (50.4/49.6)	78 (61.4)	127 (100.0)	25 (19.7)	63 (49.6)	39 (30.7)	73 (57.5)	54 (42.5)	13 (10.2)	13 (10.2)	98 (77.2)	28 (22.0)
Previously used laxatives														
Osmotic/saline laxatives only	69.0 (12.9)	53.7 (11.2)	202/172 (54.0/46.0)	245 (65.5)	374 (100.0)	88 (23.5)	213 (57.0)	73 (19.5)	259 (69.3)	115 (30.7)	25 (6.7)	18 (4.8)	234 (62.6)	62 (16.6)
Stimulant laxatives only	69.8 (12.2)	52.0 (10.5)	53/51 (51.0/49.0)	71 (68.3)	104 (100.0)	18 (17.3)	65 (62.5)	21 (20.2)	69 (66.3)	35 (33.7)	9 (8.7)	5 (4.8)	72 (69.2)	20 (19.2)
Others	68.4 (12.8)	53.6 (11.0)	153/116 (56.9/43.1)	202 (75.1)	269 (100.0)	46 (17.1)	153 (56.9)	70 (26.0)	165 (61.3)	104 (38.7)	34 (12.6)	18 (6.7)	199 (74.0)	67 (24.9)
Nothing	69.0 (13.2)	54.0 (11.1)	135/71 (65.5/34.5)	138 (67.0)	206 (100.0)	59 (28.6)	54 (26.2)	93 (45.1)	152 (73.8)	53 (25.7)	23 (11.2)	15 (7.3)	117 (56.8)	33 (16.0)
Concomitant laxatives														
Osmotic/saline laxatives only	69.4 (12.9)	53.7 (11.3)	196/168 (53.8/46.2)	244 (67.0)	364 (100.0)	94 (25.8)	196 (53.8)	74 (20.3)	255 (70.1)	109 (29.9)	21 (5.8)	13 (3.6)	216 (59.3)	54 (14.8)
Stimulant laxatives only	69.3 (12.7)	52.2 (10.5)	53/37 (58.9/41.1)	65 (72.2)	90 (100.0)	18 (20.0)	52 (57.8)	20 (22.2)	54 (60.0)	36 (40.0)	9 (10.0)	5 (5.6)	57 (63.3)	14 (15.6)
Others	68.2 (12.3)	53.7 (10.8)	209/148 (58.5/41.5)	260 (72.8)	357 (100.0)	59 (16.5)	197 (55.2)	101 (28.3)	236 (66.1)	120 (33.6)	45 (12.6)	27 (7.6)	269 (75.4)	82 (23.0)
Nothing	69.0 (14.1)	53.5 (11.5)	85/57 (59.9/40.1)	87 (61.3)	142 (100.0)	40 (28.2)	40 (28.2)	62 (43.7)	100 (70.4)	42 (29.6)	16 (11.3)	11 (7.7)	80 (56.3)	32 (22.5)

Supplementary Table A2

**Table 6:** Seriousness, time of onset, outcome at two to less than four weeks following diarrhoea and other adverse drug reactions, and time to recovery or remission ADR, adverse drug reaction.

Category	Type of ADR	n (%)		Seriousness		Time of onset								Outcome							Time to recovery or remission			
				Serious	Non-serious	<1 week	1–<2 weeks	2–<4 weeks	4–<6 weeks	6–<8 weeks	8–<10 weeks	10–<12 weeks	≥12 weeks	Recovered	Recovering	Recovered + recovering	Not recovered	Sequelae	Death	Unknown	1-3 days	4-7 days	8-14 days	≥15 days
Total	Diarrhoea	107 (100)		2 (1.9)	105 (98.1)	64 (59.8)	15 ‌(14.0)	18 (16.8)	5 (4.7)	2 (1.9)	2 (1.9)	0 (0.0)	1 (0.9)	77 (72.0)	24 (22.4)	101 (94.4)	3 (2.8)	0 (0.0)	0 (0.0)	3 (2.8)	43 (40.2)	26 (24.3)	15 (14.0)	17 (15.9)
	Other	38 (100)		7 (18.4)	31 (81.6)	16 (42.1)	8 (21.1)	8 (21.1)	4 (10.5)	1 (2.6)	1 (2.6)	0 (0.0)	0 (0.0)	18 (47.4)	16 (42.1)	34 (89.5)	3 (7.9)	0 (0.0)	0 (0.0)	1 (2.6)	11 (28.9)	7 (18.4)	8 (21.1)	8 (21.1)
Age (years)																								
<75	Diarrhoea	74 (100)		1 (1.4)	73 (98.6)	43 (58.1)	9 (12.2)	14 (18.9)	4 (5.4)	2 (2.7)	1 (1.4)	0 (0.0)	1 (1.4)	54 (73.0)	15 (20.3)	69 (93.2)	2 (2.7)	0 (0.0)	0 (0.0)	3 (4.1)	30 (40.5)	16 (21.6)	12 (16.2)	11 (14.9)
	Other	27 (100)		4 (14.8)	23 (85.2)	10 (37.0)	6 (22.2)	7 (25.9)	3 (11.1)	1 (3.7)	0 (0.0)	0 (0.0)	0 (0.0)	13 (48.1)	12 (44.4)	25 (92.6)	2 (7.4)	0 (0.0)	0 (0.0)	0 (0.0)	7 (25.9)	7 (25.9)	4 (14.8)	7 (25.9)
≥75	Diarrhoea	33 (100)		1 (3.0)	32 (97.0)	21 (63.6)	6 (18.2)	4 (12.1)	1 (3.0)	0 (0.0)	1 (3.0)	0 (0.0)	0 (0.0)	23 (69.7)	9 (27.3)	32 (97.0)	1 (3.0)	0 (0.0)	0 (0.0)	0 (0.0)	13 (39.4)	10 (30.3)	3 (9.1)	6 (18.2)
	Other	11 (100)		3 (27.3)	8 (72.7)	6 (54.5)	2 (18.2)	1 (9.1)	1 (9.1)	0 (0.0)	1 (9.1)	0 (0.0)	0 (0.0)	5 (45.5)	4 (36.4)	9 (81.8)	1 (9.1)	0 (0.0)	0 (0.0)	1 (9.1)	4 (36.4)	0 (0.0)	4 (36.4)	1 (9.1)
Performance status (PS)																								
0-2	Diarrhoea	78 (100)	2 (2.6)		76 (97.4)	46 (59.0)	10 (12.8)	15 (19.2)	3 (3.8)	2 (2.6)	1 (1.3)	0 (0.0)	1 (1.3)	57 (73.1)	17 (21.8)	74 (94.9)	1 (1.3)	0 (0.0)	0 (0.0)	3 (3.8)	29 (37.2)	17 (21.8)	13 (16.7)	15 (19.2)
	Other	30 (100)	5 (16.7)		25 (83.3)	12 (40.0)	7 (23.3)	7 (23.3)	3 (10.0)	1 (3.3)	0 (0.0)	0 (0.0)	0 (0.0)	15 (50.0)	12 (40.0)	27 (90.0)	2 (6.7)	0 (0.0)	0 (0.0)	1 (3.3)	9 (30.0)	5 (16.7)	6 (20.0)	7 (23.3)
3-4	Diarrhoea	29 (100)	0 (0.0)		29 (100)	18 (62.1)	5 (17.2)	3 (10.3)	2 (6.9)	0 (0.0)	1 (3.4)	0 (0.0)	0 (0.0)	20 (69.0)	7 (24.1)	27 (93.1)	2 (6.9)	0 (0.0)	0 (0.0)	0 (0.0)	14 (48.3)	9 (31.0)	2 (6.9)	2 (6.9)
	Other	8 (100)	2 (25.0)		6 (75.0)	4 (50.0)	1 (12.5)	1 (12.5)	1 (12.5)	0 (0.0)	1 (12.5)	0 (0.0)	0 (0.0)	3 (37.5)	4 (50.0)	7 (87.5)	1 (12.5)	0 (0.0)	0 (0.0)	0 (0.0)	2 (25.0)	2 (25.0)	2 (25.0)	1 (12.5)
Severity of constipation before naldemedine dosing																								
Mild	Diarrhoea	20 (100)	1 (5.0)		19 (95.0)	9 (45.0)	6 (30.0)	3 (15.0)	0 (0.0)	2 (10.0)	0 (0.0)	0 (0.0)	0 (0.0)	14 (70.0)	6 (30.0)	20 (100)	0 (0.0)	0 (0.0)	0 (0.0)	0 (0.0)	8 (40.0)	3 (15.0)	5 (25.0)	4 (20.0)
	Other	14 (100)	4 (28.6)		10 (71.4)	6 (42.9)	4 (28.6)	2 (14.3)	1 (7.1)	1 (7.1)	0 (0.0)	0 (0.0)	0 (0.0)	5 (35.7)	9 (64.3)	14 (100)	0 (0.0)	0 (0.0)	0 (0.0)	0 (0.0)	3 (21.4)	2 (14.3)	5 (35.7)	4 (28.6)
Moderate	Diarrhoea	60 (100)	0 (0.0)		60 (100)	40 (66.7)	9 (15.0)	6 (10.0)	3 (5.0)	0 (0.0)	1 (1.7)	0 (0.0)	1 (1.7)	46 (76.7)	11 (18.3)	57 (95.0)	1 (1.7)	0 (0.0)	0 (0.0)	2 (3.3)	23 (38.3)	16 (26.7)	7 (11.7)	11 (18.3)
	Other	15 (100)	2 (13.3)		13 (86.7)	7 (46.7)	3 (20.0)	3 (20.0)	2 (13.3)	0 (0.0)	0 (0.0)	0 (0.0)	0 (0.0)	7 (46.7)	5 (33.3)	12 (80.0)	2 (13.3)	0 (0.0)	0 (0.0)	1 (6.7)	4 (26.7)	2 (13.3)	2 (13.3)	4 (26.7)
Severe	Diarrhoea	17 (100)	0 (0.0)		17 (100)	12 (70.6)	0 (0.0)	4 (23.5)	0 (0.0)	0 (0.0)	1 (5.9)	0 (0.0)	0 (0.0)	12 (70.6)	4 (23.5)	16 (94.1)	1 (5.9)	0 (0.0)	0 (0.0)	0 (0.0)	12 (70.6)	2 (11.8)	1 (5.9)	1 (5.9)
	Other	8 (100)	1 (12.5)		7 (87.5)	3 (37.5)	1 (12.5)	3 (37.5)	0 (0.0)	0 (0.0)	1 (12.5)	0 (0.0)	0 (0.0)	6 (75.0)	1 (12.5)	7 (87.5)	1 (12.5)	0 (0.0)	0 (0.0)	0 (0.0)	3 (37.5)	3 (37.5)	1 (12.5)	0 (0.0)
Brain metastasis																								
No	Diarrhoea	102 (100)	2 (2.0)		100 (98.0)	60 (58.8)	15 (14.7)	18 (17.6)	4 (3.9)	2 (2.0)	2 (2.0)	0 (0.0)	1 (1.0)	74 (72.5)	23 (22.5)	97 (95.1)	3 (2.9)	0 (0.0)	0 (0.0)	2 (2.0)	40 (39.2)	25 (24.5)	15 (14.7)	17 (16.7)
	Other	32 (100)	7 (21.9)		25 (78.1)	15 (46.9)	7 (21.9)	5 (15.6)	3 (9.4)	1 (3.1)	1 (3.1)	0 (0.0)	0 (0.0)	17 (53.1)	13 (40.6)	30 (93.8)	1 (3.1)	0 (0.0)	0 (0.0)	1 (3.1)	10 (31.3)	6 (18.8)	7 (21.9)	7 (21.9)
Yes	Diarrhoea	5 (100)	0 (0.0)		5 (100)	4 (80.0)	0 (0.0)	0 (0.0)	1 (20.0)	0 (0.0)	0 (0.0)	0 (0.0)	0 (0.0)	3 (60.0)	1 (20.0)	4 (80.0)	0 (0.0)	0 (0.0)	0 (0.0)	1 (20.0)	3 (60.0)	1 (20.0)	0 (0.0)	0 (0.0)
	Other	6 (100)	0 (0.0)		6 (100)	1 (16.7)	1 (16.7)	3 (50.0)	1 (16.7)	0 (0.0)	0 (0.0)	0 (0.0)	0 (0.0)	1 (16.7)	3 (50.0)	4 (66.7)	2 (33.3)	0 (0.0)	0 (0.0)	0 (0.0)	1 (16.7)	1 (16.7)	1 (16.7)	1 (16.7)
Anticancer drug treatment																								
No	Diarrhoea	51 (100)	0 (0.0)		51 (100)	36 (70.6)	4 (7.8)	7 (13.7)	2 (3.9)	1 (2.0)	0 (0.0)	0 (0.0)	1 (2.0)	36 (70.6)	13 (25.5)	49 (96.1)	1 (2.0)	0 (0.0)	0 (0.0)	1 (2.0)	23 (45.1)	14 (27.5)	6 (11.8)	6 (11.8)
	Other	19 (100)	3 (15.8)		16 (84.2)	10 (52.6)	4 (21.1)	3 (15.8)	1 (5.3)	1 (5.3)	0 (0.0)	0 (0.0)	0 (0.0)	11 (57.9)	8 (42.1)	19 (100)	0 (0.0)	0 (0.0)	0 (0.0)	0 (0.0)	6 (31.6)	6 (31.6)	3 (15.8)	4 (21.1)
Yes	Diarrhoea	56 (100)	2 (3.6)		54 (96.4)	28 (50.0)	11 (19.6)	11 (19.6)	3 (5.4)	1 (1.8)	2 (3.6)	0 (0.0)	0 (0.0)	41 (73.2)	11 (19.6)	52 (92.9)	2 (3.6)	0 (0.0)	0 (0.0)	2 (3.6)	20 (35.7)	12 (21.4)	9 (16.1)	11 (19.6)
	Other	19 (100)	4 (21.1)		15 (78.9)	6 (31.6)	4 (21.1)	5 (26.3)	3 (15.8)	0 (0.0)	1 (5.3)	0 (0.0)	0 (0.0)	7 (36.8)	8 (42.1)	15 (78.9)	3 (15.8)	0 (0.0)	0 (0.0)	1 (5.3)	5 (26.3)	1 (5.3)	5 (26.3)	4 (21.1)
Opioid analgesics used when naldemedine was started																								
Fentanyl only	Diarrhoea	5 (100)	0 (0.0)		5 (100)	5 (100)	0 (0.0)	0 (0.0)	0 (0.0)	0 (0.0)	0 (0.0)	0 (0.0)	0 (0.0)	4 (80.0)	1 (20.0)	5 (100)	0 (0.0)	0 (0.0)	0 (0.0)	0 (0.0)	2 (40.0)	1 (20.0)	1 (20.0)	1 (20.0)
	Other	1 (100)	0 (0.0)		1 (100)	0 (0.0)	0 (0.0)	1 (100)	0 (0.0)	0 (0.0)	0 (0.0)	0 (0.0)	0 (0.0)	1 (100)	0 (0.0)	1 (100)	0 (0.0)	0 (0.0)	0 (0.0)	0 (0.0)	0 (0.0)	0 (0.0)	1 (100)	0 (0.0)
Only strong opioids other than fentanyl	Diarrhoea	72 (100)	2 (2.8)		70 (97.2)	43 (59.7)	10 (13.9)	10 (13.9)	5 (6.9)	1 (1.4)	2 (2.8)	0 (0.0)	1 (1.4)	50 (69.4)	18 (25.0)	68 (94.4)	2 (2.8)	0 (0.0)	0 (0.0)	2 (2.8)	30 (41.7)	16 (22.2)	10 (13.9)	12 (16.7)
	Other	29 (100)	5 (17.2)		24 (82.8)	14 (48.3)	5 (17.2)	5 (17.2)	3 (10.3)	1 (3.4)	1 (3.4)	0 (0.0)	0 (0.0)	12 (41.4)	13 (44.8)	25 (86.2)	3 (10.3)	0 (0.0)	0 (0.0)	1 (3.4)	8 (27.6)	6 (20.7)	6 (20.7)	5 (17.2)
Weak opioids only	Diarrhoea	12 (100)	0 (0.0)		12 (100)	8 (66.7)	0 (0.0)	4 (33.3)	0 (0.0)	0 (0.0)	0 (0.0)	0 (0.0)	0 (0.0)	7 (58.3)	4 (33.3)	11 (91.7)	0 (0.0)	0 (0.0)	0 (0.0)	1 (8.3)	4 (33.3)	3 (25.0)	1 (8.3)	3 (25.0)
	Other	2 (100)	0 (0.0)		2 (100)	1 (50.0)	1 (50.0)	0 (0.0)	0 (0.0)	0 (0.0)	0 (0.0)	0 (0.0)	0 (0.0)	1 (50.0)	1 (50.0)	2 (100)	0 (0.0)	0 (0.0)	0 (0.0)	0 (0.0)	1 (50.0)	0 (0.0)	0 (0.0)	1 (50.0)
Others	Diarrhoea	18 (100)	0 (0.0)		18 (100)	8 (44.4)	5 (27.8)	4 (22.2)	0 (0.0)	1 (5.6)	0 (0.0)	0 (0.0)	0 (0.0)	16 (88.9)	1 (5.6)	17 (94.4)	1 (5.6)	0 (0.0)	0 (0.0)	0 (0.0)	7 (38.9)	6 (33.3)	3 (16.7)	1 (5.6)
	Other	6 (100)	2 (33.3)		4 (66.7)	1 (16.7)	2 (33.3)	2 (33.3)	1 (16.7)	0 (0.0)	0 (0.0)	0 (0.0)	0 (0.0)	4 (66.7)	2 (33.3)	6 (100)	0 (0.0)	0 (0.0)	0 (0.0)	0 (0.0)	2 (33.3)	1 (16.7)	1 (16.7)	2 (33.3)
Previously used laxatives																								
Osmotic/saline laxatives only	Diarrhoea	43 (100)	1 (2.3)		42 (97.7)	23 (53.5)	11 (25.6)	6 (14.0)	1 (2.3)	1 (2.3)	0 (0.0)	0 (0.0)	1 (2.3)	31 (72.1)	11 (25.6)	42 (97.7)	1 (2.3)	0 (0.0)	0 (0.0)	0 (0.0)	15 (34.9)	9 (20.9)	9 (20.9)	9 (20.9)
	Other	15 (100)	2 (13.3)		13 (86.7)	10 (66.7)	1 (6.7)	3 (20.0)	1 (6.7)	0 (0.0)	0 (0.0)	0 (0.0)	0 (0.0)	8 (53.3)	7 (46.7)	15 (100)	0 (0.0)	0 (0.0)	0 (0.0)	0 (0.0)	4 (26.7)	3 (20.0)	6 (40.0)	2 (13.3)
Stimulant laxatives only	Diarrhoea	8 (100)	0 (0.0)		8 (100)	5 (62.5)	2 (25.0)	0 (0.0)	0 (0.0)	1 (12.5)	0 (0.0)	0 (0.0)	0 (0.0)	7 (87.5)	1 (12.5)	8 (100)	0 (0.0)	0 (0.0)	0 (0.0)	0 (0.0)	5 (62.5)	2 (25.0)	0 (0.0)	1 (12.5)
	Other	2 (100)	0 (0.0)		2 (100)	0 (0.0)	1 (50.0)	1 (50.0)	0 (0.0)	0 (0.0)	0 (0.0)	0 (0.0)	0 (0.0)	1 (50.0)	1 (50.0)	2 (100)	0 (0.0)	0 (0.0)	0 (0.0)	0 (0.0)	1 (50.0)	1 (50.0)	0 (0.0)	0 (0.0)
Others	Diarrhoea	35 (100)	0 (0.0)		35 (100)	27 (77.1)	2 (5.7)	4 (11.4)	2 (5.7)	0 (0.0)	0 (0.0)	0 (0.0)	0 (0.0)	27 (77.1)	7 (20.0)	34 (97.1)	0 (0.0)	0 (0.0)	0 (0.0)	1 (2.9)	14 (40.0)	11 (31.4)	4 (11.4)	5 (14.3)
	Other	13 (100)	1 (7.7)		12 (92.3)	3 (23.1)	5 (38.5)	4 (30.8)	0 (0.0)	1 (7.7)	0 (0.0)	0 (0.0)	0 (0.0)	6 (46.2)	4 (30.8)	10 (76.9)	2 (15.4)	0 (0.0)	0 (0.0)	1 (7.7)	3 (23.1)	3 (23.1)	2 (15.4)	2 (15.4)
Nothing	Diarrhoea	21 (100)	1 (4.8)		20 (95.2)	9 (42.9)	0 (0.0)	8 (38.1)	2 (9.5)	0 (0.0)	2 (9.5)	0 (0.0)	0 (0.0)	12 (57.1)	5 (23.8)	17 (81.0)	2 (9.5)	0 (0.0)	0 (0.0)	2 (9.5)	9 (42.9)	4 (19.0)	2 (9.5)	2 (9.5)
	Other	8 (100)	4 (50.0)		4 (50.0)	3 (37.5)	1 (12.5)	0 (0.0)	3 (37.5)	0 (0.0)	1 (12.5)	0 (0.0)	0 (0.0)	3 (37.5)	4 (50.0)	7 (87.5)	1 (12.5)	0 (0.0)	0 (0.0)	0 (0.0)	3 (37.5)	0 (0.0)	0 (0.0)	4 (50.0)
Concomitant laxatives																								
Osmotic/saline laxatives only	Diarrhoea	40 (100)	1 (2.5)		39 (97.5)	22 (55.0)	8 (20.0)	7 (17.5)	2 (5.0)	0 (0.0)	0 (0.0)	0 (0.0)	1 (2.5)	28 (70.0)	11 (27.5)	39 (97.5)	0 (0.0)	0 (0.0)	0 (0.0)	1 (2.5)	12 (30.0)	8 (20.0)	9 (22.5)	10 (25.0)
	Other	16 (100)	2 (12.5)		14 (87.5)	10 (62.5)	1 (6.3)	3 (18.8)	2 (12.5)	0 (0.0)	0 (0.0)	0 (0.0)	0 (0.0)	9 (56.3)	7 (43.8)	16 (100)	0 (0.0)	0 (0.0)	0 (0.0)	0 (0.0)	4 (25.0)	3 (18.8)	6 (37.5)	3 (18.8)
Stimulant laxatives only	Diarrhoea	7 (100)	0 (0.0)		7 (100)	4 (57.1)	1 (14.3)	0 (0.0)	0 (0.0)	2 (28.6)	0 (0.0)	0 (0.0)	0 (0.0)	6 (85.7)	1 (14.3)	7 (100)	0 (0.0)	0 (0.0)	0 (0.0)	0 (0.0)	5 (71.4)	1 (14.3)	0 (0.0)	1 (14.3)
	Other	2 (100)	0 (0.0)		2 (100)	0 (0.0)	1 (50.0)	1 (50.0)	0 (0.0)	0 (0.0)	0 (0.0)	0 (0.0)	0 (0.0)	1 (50.0)	1 (50.0)	2 (100)	0 (0.0)	0 (0.0)	0 (0.0)	0 (0.0)	1 (50.0)	1 (50.0)	0 (0.0)	0 (0.0)
Others	Diarrhoea	44 (100)	0 (0.0)		44 (100)	28 (63.6)	6 (13.6)	5 (11.4)	3 (6.8)	0 (0.0)	2 (4.5)	0 (0.0)	0 (0.0)	34 (77.3)	7 (15.9)	41 (93.2)	2 (4.5)	0 (0.0)	0 (0.0)	1 (2.3)	17 (38.6)	14 (31.8)	5 (11.4)	5 (11.4)
	Other	18 (100)	4 (22.2)		14 (77.8)	4 (22.2)	6 (33.3)	4 (22.2)	2 (11.1)	1 (5.6)	1 (5.6)	0 (0.0)	0 (0.0)	7 (38.9)	7 (38.9)	14 (77.8)	3 (16.7)	0 (0.0)	0 (0.0)	1 (5.6)	5 (27.8)	3 (16.7)	2 (11.1)	4 (22.2)
Nothing	Diarrhoea	16 (100)	1 (6.3)		15 (93.8)	10 (62.5)	0 (0.0)	6 (37.5)	0 (0.0)	0 (0.0)	0 (0.0)	0 (0.0)	0 (0.0)	9 (56.3)	5 (31.3)	14 (87.5)	1 (6.3)	0 (0.0)	0 (0.0)	1 (6.3)	9 (56.3)	3 (18.8)	1 (6.3)	1 (6.3)
	Other	2 (100.0)	1 (50.0)		1 (50.0)	2 (100.0)	0 (0.0)	0 (0.0)	0 (0.0)	0 (0.0)	0 (0.0)	0 (0.0)	0 (0.0)	1 (50.0)	1 (50.0)	2 (100.0)	0 (0.0)	0 (0.0)	0 (0.0)	0 (0.0)	1 (50.0)	0 (0.0)	0 (0.0)	1 (50.0)

## References

[1] Els C, Jackson TD, Kunyk D (2017). Adverse events associated with medium- and long-term use of opioids for chronic non-cancer pain: an overview of Cochrane Reviews. Cochrane Database Syst Rev.

[2] Farmer AD, Drewes AM, Chiarioni G, De Giorgio R, O'Brien T, Morlion B, Tack J (2019). Pathophysiology and management of opioid-induced constipation: European expert consensus statement. United European Gastroenterol J.

[3] Tokoro A, Imai H, Fumita S (2019). Incidence of opioid-induced constipation in Japanese patients with cancer pain: a prospective observational cohort study. Cancer Med.

[4] Andresen V, Banerji V, Hall G, Lass A, Emmanuel AV (2018). The patient burden of opioid-induced constipation: new insights from a large, multinational survey in five European countries. United European Gastroenterol J.

[5] De Giorgio R, Zucco FM, Chiarioni G (2021). Management of opioid-induced constipation and bowel dysfunction: expert opinion of an Italian multidisciplinary panel. Adv Ther.

[6] Japanese Society for Palliative Medicine Guideline Management Committee (2023). Guidelines for Drug Therapy for Cancer Pain (2020 Edition). Guidelines for Drug Therapy for Cancer Pain (2020 Edition).

[7] Crockett SD, Greer KB, Heidelbaugh JJ, Falck-Ytter Y, Hanson BJ, Sultan S (2019). American Gastroenterological Association Institute guideline on the medical management of opioid-induced constipation. Gastroenterology.

[8] Mesía R, Virizuela Echaburu JA, Gómez J, Sauri T, Serrano G, Pujol E (2019). Opioid-induced constipation in oncological patients: new strategies of management. Curr Treat Options Oncol.

[9] Pergolizzi JV Jr, Christo PJ, LeQuang JA, Magnusson P (2020). The use of peripheral μ-opioid receptor antagonists (PAMORA) in the management of opioid-induced constipation: an update on their efficacy and safety. Drug Des Devel Ther.

[10] Rekatsina M, Paladini A, Drewes AM (2021). Efficacy and safety of peripherally acting μ-opioid receptor antagonist (PAMORAs) for the management of patients with opioid-induced constipation: a systematic review. Cureus.

[11] Kanemasa T, Koike K, Arai T (2019). Pharmacologic effects of naldemedine, a peripherally acting μ-opioid receptor antagonist, in in vitro and in vivo models of opioid-induced constipation. Neurogastroenterol Motil.

[12] Inagaki M, Kume M, Tamura Y (2019). Discovery of naldemedine: a potent and orally available opioid receptor antagonist for treatment of opioid-induced adverse effects. Bioorg Med Chem Lett.

[13] Nee J, Zakari M, Sugarman MA (2018). Efficacy of treatments for opioid-induced constipation: systematic review and meta-analysis. Clin Gastroenterol Hepatol.

[14] Dzierżanowski T, Mercadante S (2022). Constipation in cancer patients - an update of clinical evidence. Curr Treat Options Oncol.

[15] (2022). European Medicines Agency. Rizmoic. https://www.ema.europa.eu/en/medicines/human/EPAR/rizmoic.

[16] Markham A (2017). Naldemedine: first global approval. Drugs.

[17] (2021). Taiwan Food and Drug Administration. Assessment report: naldemedine tosylate. https://www.fda.gov.tw/tc/includes/GetFile.ashx?id=f637738690978327922&type=2&cid=39298.

[18] Hale M, Wild J, Reddy J, Yamada T, Arjona Ferreira JC (2017). Naldemedine versus placebo for opioid-induced constipation (COMPOSE-1 and COMPOSE-2): two multicentre, phase 3, double-blind, randomised, parallel-group trials. Lancet Gastroenterol Hepatol.

[19] Katakami N, Harada T, Murata T (2017). Randomized phase III and extension studies of naldemedine in patients with opioid-induced constipation and cancer. J Clin Oncol.

[20] Saito Y, Yokota T, Arai M, Tada Y, Sumitani M (2019). Naldemedine in Japanese patients with opioid-induced constipation and chronic noncancer pain: open-label phase III studies. J Pain Res.

[21] Webster LR, Nalamachu S, Morlion B, Reddy J, Baba Y, Yamada T, Arjona Ferreira JC (2018). Long-term use of naldemedine in the treatment of opioid-induced constipation in patients with chronic noncancer pain: a randomized, double-blind, placebo-controlled phase 3 study. Pain.

[22] Takata K, Nakazawa M, Honda K, Hashimoto S (2022). Post-marketing surveillance of the safety and effectiveness of naldemedine in the management of opioid-induced constipation in patients with cancer pain in Japan. Support Care Cancer.

[23] Osaka I, Ishiki H, Yokota T, Tada Y, Sato H, Okamoto M, Satomi E (2019). Safety and efficacy of naldemedine in cancer patients with opioid-induced constipation: a pooled, subgroup analysis of two randomised controlled studies. ESMO Open.

[24] Katakami N, Harada T, Murata T (2018). Randomized phase III and extension studies: efficacy and impacts on quality of life of naldemedine in subjects with opioid-induced constipation and cancer. Ann Oncol.

[25] Wilhelm I, Molnár J, Fazakas C, Haskó J, Krizbai IA (2013). Role of the blood-brain barrier in the formation of brain metastases. Int J Mol Sci.

[26] Iijima K, Arai H, Akishita M (2021). Toward the development of a vibrant, super-aged society: the future of medicine and society in Japan. Geriatr Gerontol Int.

[27] Kirkova J, Rybicki L, Walsh D, Aktas A (2012). Symptom prevalence in advanced cancer: age, gender, and performance status interactions. Am J Hosp Palliat Care.

[28] Akbarali HI, Muchhala KH, Jessup DK, Cheatham S (2022). Chemotherapy induced gastrointestinal toxicities. Adv Cancer Res.

[29] Hashizume J, Shiojiri K, Ryu E (2021). Analysis of predictive factors for diarrhea after the administration of naldemedine. Biol Pharm Bull.

[30] Ghoshal A (2020). Fentanyl, morphine, and opioid-induced constipation in patients with cancer-related pain. Indian J Palliat Care.

